# Survivor Guilt: A Cognitive Approach

**DOI:** 10.1017/S1754470X21000246

**Published:** 2021-09-16

**Authors:** Hannah Murray, Yasmin Pethania, Evelina Medin

**Affiliations:** University of Oxford; University of Surrey; Royal Holloway, University of London

**Keywords:** PTSD, guilt, trauma, cognitive therapy, CBT

## Abstract

Survivor guilt is a common experience following traumatic events in which others have died. However, little research has addressed the phenomenology of survivor guilt, nor has the issue been conceptualised using contemporary psychological models which would help guide clinicians in effective treatment approaches for this distressing problem. This paper summarises the current survivor guilt research literature and psychological models from related areas, such as posttraumatic stress disorder, moral injury and traumatic bereavement. Based on this literature, a preliminary cognitive approach to survivor guilt is proposed. A cognitive conceptualisation is described, and used as a basis to suggest potential treatment interventions for survivor guilt. Both the model and treatment strategies require further detailed study and empirical validation, but provide testable hypotheses to stimulate further research in this area.

## Introduction

Survivor guilt is a commonly-used term in both clinical descriptions and lay language, and has been identified in a range of trauma-exposed populations, often linked to more severe post-traumatic mental health consequences (e.g. Murray, 2018). Guilt is a self-conscious affect and moral emotion characterised by negative self-evaluation (Tangney, & Dearing, 2002; Tangney et al., 2007) and is a common post-traumatic experience. Survivor guilt typically arises in people who have been exposed to, or witnessed, death and have stayed alive (Lifton, 1980), leading to emotional distress and negative self-appraisal. Often, survivors feel responsible for the death or injury of others, even when they had no real power or influence in the situation (Tangney, & Dearing, 2002).

Survivor guilt was once considered a symptom of Post-Traumatic Stress Disorder, according to DSM-III (Diagnostic and Statistical Manual of Mental Disorders, 3^rd^ edition, American Psychiatric Association, 1980), reflecting the influence at the time of research focused on Vietnam war veterans, who reported high levels of survivor guilt (e.g. Hendin & Haas, 1991). It was listed as an associated symptom of PTSD in DSM-IV-TR (APA, 2000), then removed in the most recent diagnostic criteria, DSM-5 (APA, 2013). Despite its previous diagnostic importance, the experience has been rarely studied systematically. Existing theoretical accounts are primarily psychoanalytic, derived from observational studies, and not empirically tested. Very few treatment studies have ever been published.

This paper briefly summarises the current literature on survivor guilt and related fields. This will be used as a basis for outlining a cognitive conceptualisation of survivor guilt which can generate testable hypotheses about the origins and maintenance of the problem, as well as ideas for intervention strategies. It is hoped that it will also provide clinicians working with survivors of traumatic events with the tools to recognise survivor guilt, and with a starting point for intervention.

The conceptualisation outlined in this paper draws on work by other theorists in related fields such as cognitive models of PTSD (particularly Ehlers & Clark, 2000, and Resick & Schnicke, 1992; 1993), trauma-related guilt (Kubany & Manke, 1995; Lee et al., 2001), traumatic bereavement (e.g. Boelen et al., 2006) and moral injury (Litz et al., 2009). Rather than aiming to replace these existing models, this article aims to apply understanding generated from such frameworks to the experience of survivor guilt.

## Prevalence literature

Survivor guilt has been documented in therapeutic writing for centuries. Freud, after the death of his father, noted his own experience of ‘self-reproach that regularly sets in among the survivors’ (Freud, 1895/1985). Niederland (1968) wrote extensively about survivor guilt in Holocaust survivors, coining the term ‘survivor syndrome’. A similar pattern of pathology was noted in Lifton’s (1976) detailed observations of survivors of the Hiroshima attack.

More recently, survivor guilt has been reported in a wide range of traumatised groups, including refugee populations (Bemak & Chung, 2017), military veterans (Currier et al., 2015), survivors of terrorist attacks (Mallimson, 2005), HIV-negative gay men (Wayment et al., 1995), grandparents who had lost a grandchild (Fry, 1997), and survivors of mass-casualty accidents (Hull et al., 2002). Prevalence studies have shown that survivor guilt is a common experience in such groups. Okulate and Jones (2006) found survivor guilt in 38% of their sample of Nigerian soldiers, a similar proportion to the 46% that Hendin and Haas (1991) found in veterans of the Vietnam war. Survivor guilt has also been recorded in medical populations, for example in 55% of lung cancer survivors (Perloff et al., 2019). It also seems a long-lasting experience. In survivors of a mass-casualty maritime accident, 61% reported survivor guilt 30 months after the incident (Joseph et al., 1993) and, ten years after the Piper Alpha oil platform disaster, Hull et al. (2002) found rates of survivor guilt were 31%.

This article will address survivor guilt following situations in which other people have died, but it is worth noting that some studies have used the term more broadly. For example, some have used ‘survivor guilt’ to describe guilt experienced by survivors of non-life-threatening events, such as redundancies in the workplace (e.g. Brockner et al., 1985) and first-generation university students, who grew up in low-income households, and have surpassed the academic achievements of others in their family and community (Austin et al., 2009; Piorkowski, 1983). Here, the sense of having benefitted more than (or at the expense of) others, has parallels to surviving fatal traumas but also important differences.

Prevalence data on survivor guilt has generally been sourced from specific cohorts of trauma survivors. However, it was also found to be common amongst those attending a UK trauma clinic (Murray, 2018); where nearly 40% of clients had experienced a trauma in which someone died, and 90% of those reported guilt about survival, mostly at severe levels. The experience of survivor guilt was associated with more severe PTSD symptoms, a finding also reported by Hull et al. (2002) and Wang et al. (2018). Furthermore, Hendin and Haas (1991) reported that survivor guilt was associated with post-service suicide attempts in a military veteran population. The high prevalence of survivor guilt in clinical populations, and its association with PTSD severity and suicide, suggests it may be a relevant target for clinical intervention.

## Theoretical considerations

### Theories of survivor guilt

Despite the high prevalence of survivor guilt in traumatised groups, few theoretical models have been developed to guide treatment. Psychoanalytic accounts, such as those by Niederland (1968) following the Holocaust, viewed survivor guilt as a psychic conflict occurring when the inmates of concentration camps had identified with the aggressor (i.e. the guards), leading to an unconscious sense that they had betrayed their fellow captives who died. Lifton (1978) noted something similar in Hiroshima survivors, describing the frequent sense that another’s life had been sacrificed at the expense of their own.

Equity theory (e.g. Walster et al., 1973), which suggests that people prefer outcomes which are fair and deserving, may be linked to survivor guilt. Baumeister et al. (1994) suggested that guilt can occur as a result of positive inequity, when people feel they have benefitted unfairly. The function of guilt in this context is the preservation of that interpersonal relationship i.e. the beneficiary seeks to ‘even the score’ in order to prevent the deterioration of the relationship. In the case of survivor guilt, this is problematic because the victim of the inbalance is deceased so cannot be ‘compensated’ and the relationship cannot be repaired. O’Connor et al. (2000) view survivor guilt as an evolutionary strategy that developed to promote group cohesion, inhibit anti-social competition, and engage in altruistic behaviour. There is some evidence that survivor guilt leads to helping behaviours. For example, Valent (1984) reported that guilt led survivors of huge bush fires in Australia to share their homes with those who had been made homeless. Wang et al. (2018) found that survivor guilt following an earthquake had a positive effect on social support, which in turn predicted post-traumatic growth, suggesting that those who felt guilty displayed more altruistic behaviour.

A more recent theoretical account of survivor guilt by Pethania et al. (2018), based on interviews with PTSD sufferers, found that survivors were caught in a continual battle to make sense of their survival, leading to persistent guilt and feelings of disentitlement to life. Survivors commonly reported a sense of wanting to repair or make amends in some way for surviving, but few had found a means to do so. Those who had seemed able to break out of the constant rumination, but were vulnerable to returning to it when their attempts to ‘work off’ the guilt felt insufficient. Although yet to be further tested, this model provides an initial perspective on the phenomenology of survivor guilt based on a more systematic analysis than the observational accounts previously described.

### Relevant cognitive-behavioural models

As yet, there are no published cognitive-behavioural conceptualisations of survivor guilt. Such models may be particularly appropriate, since accounts of survivor guilt often describe beliefs about the unfairness of survival, or being less worthy than those who died (e.g. Perloff et al., 2019; Pethania et al., 2018).

CBT models from related areas are likely to be relevant. Ehlers and Clark’s (2000) cognitive theory of PTSD conceptualises PTSD as arising from appraisals of the traumatic event which lead to an ongoing sense of current threat. For example, where an individual believes that they did not cope adequately during a traumatic event, they may doubt their ability to cope with future danger, and feel more afraid. The cognitive model also acknowledges the broad range of emotions that individuals experience during and after traumatic events. Although fear was once assumed to be the primary emotion underlying PTSD, research has shown that other emotional experiences such as guilt and shame are both common and problematic (Holmes et al., 2005), and are linked to idiosyncratic appraisals made by individuals during or after the traumatic event (Ehlers & Clark, 2000). Survivor guilt may be one such emotional experience, arising from threat appraisals such as ‘other people dying instead of me means that I have done something wrong by surviving’.

Although PTSD models are relevant, not everyone who experiences survivor guilt will meet diagnostic criteria for PTSD. For example, they may ruminate about a death but not intrusively re-experience it. Also, the event may not meet Criterion A for a PTSD diagnosis (which, according to DSM-5 must involve either witnessing the death or, if indirectly experienced or learnt about, the death must have been violent or accidental). For example, survivors of the COVID-19 pandemic (which, at the time of writing, has infected hundreds of millions of people worldwide, and killed over four million), may not develop PTSD but feel survivor guilt nonetheless. A model for survivor guilt should, therefore, be applicable to those with or without PTSD.

Research into other forms of trauma-related guilt is also relevant. Guilt following traumatic experiences is a common experience, and is associated with increased severity of PTSD (Kubany et al., 1995) and other maladaptive health outcomes (Li et al., 2014). Cognitive models addressing trauma-related guilt, such as Lee et al. (2001), highlight the centrality of guilt, shame and/or humiliation in the experience of many trauma survivors, and the importance of pre-existing schemas in conceptualising and treating the problem.

Approaches to alleviate guilt following trauma (e.g. Kubany & Manke, 2005) are often very effective in addressing the distorted cognitions which lead to guilt, for example that the sufferer could somehow have prevented the trauma. However, cognitive models of guilt often focus on appraisals relating to a sense of personal responsibility for a trauma, such as beliefs about preventability or perceived wrongdoing. Survivor guilt, however, often exists in the absence of a perception of responsibility. Survivors often know that there is nothing they could have done to prevent the death of another, but feel guilty nonetheless.

A relevant distinction between content and existential guilt was suggested by Matsakis (1999). Content survivor guilt occurs when the survivor believes that something they did or did not do led to the death of another, whereas existential guilt relates merely to surviving, even when the survivor knows they were not to blame for the death(s). We would suggest that cognitive interventions for guilt, such as those proposed by Kubany and Manke (2005) are likely to be effective when treating content survivor guilt, and that further consideration of how to work with existential survivor guilt is needed.

Guilt is one of the dominant emotions for survivors of morally injurious events, i.e. those who have perpetrated or witnessed acts that transgress their moral code. Litz et al. (2009) argue that contemporary theoretical models of PTSD do not adequately explain or provide clinical guidance for moral injury. They propose a model in which the experience of moral injury results from a dissonance between the event and pre-existing beliefs and assumptions about how the world operates, leading to a sense of guilt, shame, and anxiety. This is maintained by withdrawal from others, and failure to self-forgive. Recent treatment approaches to moral injury (e.g. Murray, & Ehlers, 2021) suggest addressing excessively negative appraisals using cognitive techniques, and identifying where meanings have become generalised and extrapolated, leading to an inability to process the event within an individual’s world, self and other view. Survivor guilt is often considered one type of moral injury. Indeed, studies of the Moral Injury Questionnaire (Currier et al., 2015), have shown that a survivor guilt question is one of the most commonly endorsed items in veteran samples (Currier et al., 2015). Survival may be construed as a transgression of an individual’s morals, or an aberration in how an ethical world should operate.

In addition to the moral injury that survival can cause, many survivors will also experience a grief reaction, especially when they knew the people who died. Research into complicated grief is also therefore relevant. For example, Boelen et al. (2006) propose that complicated grief arises from a failure to integrate the loss into the autobiographical knowledge system, together with negative beliefs about the loss, and anxious and depressive avoidance strategies. It is easy to see how survivor guilt could form part of a complicated grief reaction. For example, negative beliefs about the loss may relate to the unfairness of survival (e.g. ‘they didn’t deserve to die over me’), leading to avoidance of accepting the reality of the death of others and of reminders of the loss (e.g. talking about it with others), perpetuating the sense of guilt.

### Treatment interventions

Very little treatment research has been published which specifically addresses survivor guilt. Logotherapy, developed by Frankl (1953, 2014), a Holocaust survivor himself, has been applied to survivor guilt with military veterans (Southwick et al., 2006) and first generation college students (Tate et al., 2013), although without systematic evaluation. Logotherapy has a central tenet the goal of finding meaning in suffering, so is described in these studies as a framework to help survivors make sense of their experiences.

Valent (2016) recommends recognition of survivor guilt and its manifestations as an important aspect of treating the problem, as well as the exploration of facts relating to the circumstances of the deaths. These generally reveal that the survivor has done their best, and ‘it was the circumstances, not the survivor that were irrational or bad’, paving the way for alternative, hopeful meanings. This approach would align with cognitive-behavioural approaches to addressing guilt following trauma but has not, to our knowledge, been empirically evaluated.

A small proof-of-concept trial by Murray et al. (2020) also drew on cognitive therapy techniques to address survivor guilt using imagery rescripting and found preliminary evidence this may be a useful intervention to enable survivors to access and change images and memories linked to survivor guilt. In this study, participants with survivor guilt and PTSD were asked to identify images closely linked to their survivor guilt and to change them in imagination, in whichever way they chose. This led to a reduction in distress, cognitions, and emotions related to survivor guilt, although longer-term follow-up and replication in a larger sample would be needed to further evaluate this technique.

Other treatment approaches which have been found to be highly effective for related problems, such as PTSD, for example trauma-focused cognitive behavioural therapies and eye movement desensitization and reprocessing therapy, have not yet been specifically tested in relation to survivor guilt.

## Proposed cognitive model of existential survivor guilt

**Figure 1:**
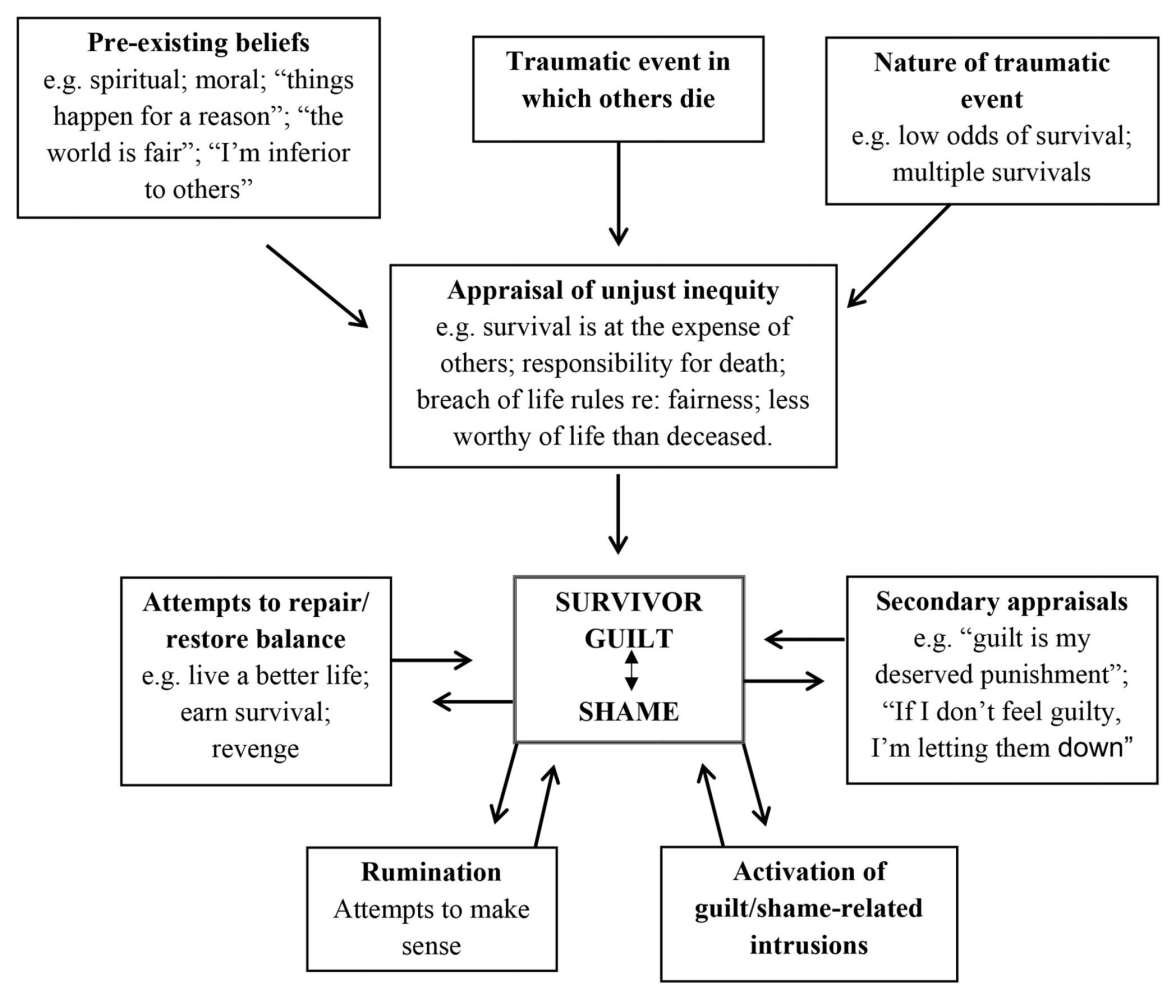
A cognitive model of survivor guilt

A cognitive understanding of survivor guilt seems apt, and we have attempted to combine the most relevant aspects of the theories and research discussed so far to propose a working model of the development and maintenance of survivor guilt (Figure 1). The cognitive model draws on established cognitive theory applied to similar problems, as well as our own clinical experience. However, this should be considered a preliminary model which requires further investigation and testing.

## Core features of survivor guilt

In keeping with equity theories of guilt, we propose that survivor guilt arises from an appraisal of unjust inequity, where the survivor perceives themselves to be an undeserving beneficiary. The inequity appraisal may be interpersonal e.g. that the person who died was more deserving of survival, or that surviving came at the expense of another’s life; or global, for example that the world is an unfair, unequal place. We suggest that inequity beliefs may include responsibility appraisals related to content survivor guilt, but in this section will focus on existential appraisals, such as survival breaching unwritten rules about life and death. These beliefs lead to the emotional experience of survivor guilt and shame where the individual feels they have done something wrong by surviving or view themselves as undeserving of their perceived benefit.

Literature often describes guilt as relating to a perceived action or inaction, while shame arises from a sense of the self as inferior or unworthy (Tangney & Dearing, 2003). Pethania et al.’s (2018) study found both guilt and shame in survivors. The two emotions reinforced each other via circular beliefs, for example ‘I didn’t deserve to survive, so I must have done something wrong; I did something wrong, so I didn’t deserve to survive’.

## Predisposing factors

We propose that several variables affect how an event is appraised. Firstly, similar to Lee et al. (2001), we suggest that pre-existing schema affect the cognitive processing of the event. These include self-schema. Where individuals have pre-existing negative self-beliefs, they may be more likely to perceive themselves as undeserving of survival. For example, someone who believes ‘I am less worthwhile than others’ may interpret their survival as unfair because ‘the other person deserved to survive more than me’, leading to feelings of unworthiness and shame. They may also believe that others will think the same (‘they wish the other person had survived instead of me’. Additionally, individuals will hold pre-existing beliefs about the world, either positive (e.g. ‘the world is a fair place’), or negative (e.g. ‘the world is a dangerous place’), which may be shattered or confirmed respectively by the event. Spiritual or religious beliefs may be brought into question. Lerner’s (1965) theory of ‘belief in a just world’ (reviewed and updated in Furnham, 2003), describes the implicit assumption that most people hold that the world ultimately operates in a fair and predictable manner, with people ‘getting what they deserve’. As Hollon and Garber (1988) describe, when a traumatic event challenges such pre-existing beliefs, instead of accommodating the new information, it is common instead to deny it, or ‘overaccommodate’ (Resick & Schnicke, 1992; 1993) by completely changing their world view e.g. to one of a completely unfair/unjust world. We propose that when pre-existing beliefs are held strongly or inflexibly, an individual will struggle to accommodate the event within their belief system, leading to a greater likelihood that an unjust inequity appraisal is made.

Other potential predisposing factors relate to the type of event experienced. We propose that survivor guilt is more likely when there has been a high number of casualties, and chances of survival were small, since this will intensify the sense of inequity. Similarly, higher rates of survivor guilt would be expected if an individual has survived multiple events in which others have died. Survivor guilt may also be more likely when people have experienced impossible choices, such as whether to give up information under torture or be executed, or escape from a burning building or stay to help others and risk dying. Finally, we predict that survivor guilt is more likely to occur when the survivor perceives that they had an equal chance of survival as those who died, or were ‘in the same boat’ (Pethania et al., 2018) as the deceased. For example, COVID-19 patients admitted to ICU who knew that many other patients on the ward died, but not them.

## Maintaining processes

We propose four main processes which maintain survivor guilt.

### 1 Attempts to restore balance

As previously described, one theory of why guilt evolved is that it promotes prosocial behaviour in groups, preserving interpersonal bonds. We suggest that in the case of survivor guilt, individuals feel a need to restore the balance of perceived inequity. However, they are often unable to do so in a satisfactory way (in part because the person who they feel has been unfairly disadvantaged has died), leading to the perpetuation of the guilty feelings.

All of the participants in Pethania et al.’s (2018) study described attempts or desires to somehow compensate for their perceived unfair benefit in surviving. In addition to prosocial activities, participants reported feeling as if they should be making more of their life and appreciating it more, although this was often hampered by the psychological distress and, for some, physical consequences of the trauma. Some participants described desire for revenge towards those they held responsible for the death, which may be viewed as an alternative form of attempted restoration of balance, but was again often impossible to achieve satisfactorily.

### 2 Rumination

A second common process amongst survivors is a continual search for meaning (described by every participant in Pethania et al.’s (2018) study), as individuals try to make sense of their survival. In some cases, this takes the form of counterfactual thinking (asking ‘what if?’ questions repeatedly). In others, the difficulty lies in attempting to answer existential questions which do not have a logical answer (e.g. ‘why them and not me?’), or an inability to accept a logical answer (e.g. ‘it was random chance’) at an emotional level.

The ongoing rumination process is driven by the sense of survivor guilt, as the survivor seeks to resolve negative emotion; but also maintains it, as the inability to ‘make sense’ of what happened fails to absolve the survivor of their perceived transgression.

### 3 Activation of guilt/shame-related intrusions

We propose that many survivors will experience intrusive memories linked to the loss event, which will be both triggered by the feeling of survivor guilt, and also lead to it, contributing to the maintenance of the problem.

In Pethania et al.’s (2018) study, some intrusive memories related to the loss event were PTSD re-experiencing symptoms of witnessing death or seeing dead bodies. Others described recurrent memories of the person alive, such as the last time they saw them, or a moment when they could have intervened to prevent the death. Some intrusions were of constructed images rather than a true memory (e.g. the person dying in pain, even when this was not witnessed). We suggest that, whether or not PTSD is present, the experience of survivor guilt will often trigger images related to the loss event. Additionally, memories and images will be triggered by reminders of the event (such as similar events in the media, anniversaries etc), intensifying the feelings of survivor guilt.

### 4 Secondary appraisals

Common amongst those with survivor guilt are secondary appraisals, or metacognitions, about guilt. For example, some of the participants in Pethania et al.’s (2018) study reported believing that guilt was a punishment for surviving, or a way of remembering those who died. It may be that the pain of experiencing survivor guilt is perceived as a way of correcting the balance when surviving has been appraised as undeserved or at the expense of another. Such appraisals will maintain survivor guilt, and interfere with therapeutic attempts to reduce it.

## Treatment implications

The proposed cognitive model of survivor guilt gives rise to a number of treatment recommendations, listed below. At present, these techniques have not been subject to empirical validation. However, they are based on other cognitive therapy interventions, adapted for this particular problem, and on our own clinical practice. These interventions can be offered as a stand-alone treatment, or within a broader CBT intervention, for example where survivor guilt presents alongside PTSD or depression.

### 1 Normalisation of survivor guilt

Providing normalising information about survivor guilt may include explaining that it is an extremely common experience after trauma and reflects the empathy that the survivor feels for others. This is an initial step in addressing metacognitive beliefs about guilt, such as the emotional reasoning thinking error ‘if I feel guilty, I must have done something wrong’.

Normalisation has the further advantage of helping to build a therapeutic alliance. This is central to most psychological interventions, but especially important where the areas being discussed are as personal and painful as survivor guilt. Throughout the intervention, the therapist takes an empathic and collaborative stance to understand the client’s experiences.

### 2 Identification and addressing inequity appraisals

The next step is to identify and address the unjust inequity appraisals which lead to survivor guilt. The goal is to help the client to find a personally meaningful explanation for their survival which allows them to accommodate the trauma within their belief system. Appraisals will be idiosyncratic, so the therapist takes time to understand the meaning of survival to the individual, using core cognitive therapy techniques such as Socratic questioning and ‘downward arrowing’.

Identification of appraisals may reveal beliefs about perceived wrongdoing. In these cases, techniques developed for trauma-related guilt in other cognitive models (Ehlers & Clark, 2000; Kubany & Manke, 1996; Lee et al., 2001; Resick & Schnicke, 1992; 1993) can be used to identify and challenge responsibility appraisals (for example, using responsibility pie charts) and thinking errors such as hindsight bias. Recognition of impossible choices (with no ‘good’ outcome) in important where relevant. Since these techniques are well documented elsewhere, they are not described in detail here.

Existential guilt, whereby the survivor feels guilty or ashamed in the absence of clear responsibility appraisals, can also be approached through consideration of alternative explanations for survival. For example, when a belief relates to the sense that the deceased somehow took the place of the survivor, the circumstances of the trauma should be carefully considered. Very often survival is a matter of chance, or related to factors outside the individual’s control (such as the survivor of the train crash who happened to sit in a certain carriage, or the soldier who was ordered to search a different building to the one which was mined). In general, the circumstances of most tragedies are that the death of one individual has not spared the life of another; deaths occur regardless of who survives.

Therapists can help clients to access and understand the implicit world views that underlie the sense that their survival is unjust. Work on inequity appraisals may need to include acknowledgment and acceptance that inequity does occur, and that some of the rules that we assume or hope operate are not consistent. This can be unsettling, as the world may seem less predictable, but the therapist can work with the client to consider alternative, flexible and realistic world views, such as ‘bad things can happen to anyone, but are very rare’, within which the experience of survival can be accommodated.

Where the event has led to the re-examination of spiritual or religious beliefs, the therapist can discuss and examine these with the client. In some cases, it may help to seek an understanding and supportive religious leader in the client’s faith to consult with them on the spiritual conflict that survival has caused.

A common belief is that the deceased was more worthy of survival, and there can be a tendency to idealise the dead (Pethania et al., 2018). These beliefs can be gently addressed through use of Socratic techniques, such as encouraging the client to view the situation from different perspectives. For example, would they judge other survivors in the same way? Exploring such beliefs often reveals pre-existing low self-esteem, and cognitive techniques to address these beliefs about the self may be required (e.g. Fennell, 1997, 1998).

One common difficulty in working with inequity appraisals is ‘head-heart lag’ (or ‘rational-emotional dissociation’; Stott, 2007), whereby the individual can see the logic and rationality of an explanation for their survival, but does not feel the corresponding emotions. For this reason, we suggest that experiential techniques such as behavioural experiments, surveys, ‘empty chair’ and imagery exercises are used when addressing appraisals. Preliminary evidence suggests that imagery rescripting (ImRs) may be a helpful intervention for survivor guilt (Murray et al., 2020). ImRs allows new beliefs to be introduced into memories by creating imaginary scenarios such as changing the ending of a memory, bringing in the older self to help the younger self, and having imagery conversations. For example, if cognitive restructuring has led a survivor to a new belief that the deceased would want them to be happy and move on with their life, this may feel more emotionally resonant if they imagine the deceased saying this to them in an imagined conversation with the deceased in imagery.

### 3 Challenging secondary appraisals that maintain guilt

Addressing inequity appraisals may reveal secondary beliefs about guilt. For example, some individuals believe that they deserve to feel guilty, or that reducing their sense of guilt will be somehow disrespect the deceased, or mean they are forgotten. This will mean that attempts to reduce guilt will feel uncomfortable or distressing.

As before, beliefs should be addressed carefully and Socratically, in the context of a strong therapeutic relationship. Useful cognitive change techniques include considering the advantages and disadvantages of feeling guilty, examination of evidence, perspective change techniques (if you had died, and the other person had not, would you believe that they deserved to feel guilty?), surveys, and behavioural experiments. Again, experiential techniques will help to strengthen any new beliefs.

For example, Jack believed that if he did not feel guilty, he would somehow be minimising the death of his brother. In therapy, we discussed whether guilt was necessary to remember a loved one, and concluded that his brother would still be much missed, loved and remembered, even without guilt. Furthermore, feeling guilty would not be what his brother would have wanted for Jack, and he imagined discussing this with his brother in imagery. The guilt had the added disadvantage of making Jack unhappy, which caused further pain to his parents. He wrote a letter to his brother, explaining how much the loss had affected him, and how much he loved him, and left it at his grave. He agreed with his parents that they would always spend his brother’s birthday together, remembering him and telling stories about him, so that he would never be forgotten. A decrease in the belief that guilt was necessary to value his brother allowed Jack to continue areas of his life that he had been avoiding, such as having relationships, developing his career, and having fun.

### 4 Addressing rumination

Rumination is a common feature of survivor guilt, often as an attempt to understand the meaning of survival, as well as counterfactual thinking about what could have been done differently to change events. Rumination is a common maintenance factor across various disorders (McLaughlin, & Nolen-Hoeksema, 2011) and CBT interventions designed to target the process of rumination are likely to be helpful for survivor guilt. These typically include a functional analysis of rumination behaviour, helping an individual to recognise when they ruminate and the effects (generally negative) it has, followed by experiments to replace rumination with alternative behaviours.

In survivor guilt, consideration of the advantages and disadvantages of rumination often reveals secondary appraisals (see previous section), such as ‘if I don’t stop thinking about it, I’m letting myself off the hook’, which will need to be addressed. Discussion will also often reveal that rumination does not usually help either the individual suffering from guilt, or the person who has died. For some individuals, this may lead to a conversation about the instinct to repair in some way for the perceived inequity (next section), which is a way of moving dwelling into positive action in the present, and for the future.

In some cases, discussion of alternatives to rumination (or other avoidance strategies) leads to the idea of acceptance. This will follow from the work (previously described) on developing a personally meaningful explanation for survival, which should reduce the need to continually search for meaning through rumination. If it continues, the therapist may need to consider whether blocking beliefs, or perhaps head-heart lag, are preventing acceptance and address them accordingly.

It is also important to note that acceptance does not suggest that the individual will be unchanged, or untroubled by the incident. Janoff-Bulman and Timko (1987) report the potential for individuals to emerge ‘sadder but wiser’ after a survival event. The therapist can, if appropriate, discuss the possibility of post-traumatic growth, although this is not to be expected for all. Some of our clients, for example, have noted that they have a greater appreciation of their loved ones after a bereavement, or a sense of heightened value in their own life. These interventions are not attempts to minimise the negative impact of survivor guilt, but to disrupt the negative repetitive cycle of rumination by encouraging an acceptance of the fact of survival, with its multi-faceted emotional experience.

### 5 Attempts to repair

Survivors often report an urge to compensate or repair in some way for their survival, conceptualised in this model as (generally frustrated) attempts to redress the imbalance created by their perceived undue and undeserved benefit at the expense of others.

There is not yet clear evidence whether encouraging outlets for the repair urge in therapy is helpful, or whether it maintains the problem by providing indirect support for the belief that the person has unfairly benefitted and should therefore compensate. Pethania et al. (2018) noted that the participants in her study who were attempting to repair (one by setting up a charity to help other survivors, another by studying for a caregiving profession) did experience less rumination, but both continued to feel guilty. Southwick et al. (2006) included repair activities, often in the form of voluntary work, in their treatment for military veterans, and report various successful cases, such as a veteran whose survivor guilt was assuaged by setting up a scholarship fund in the name of his friend who had died.

Although further research is needed, the cognitive model outlined in this paper would suggest that repair activities will maintain survivor guilt while the underlying belief remains unchallenged, as it is unlikely that the survivor will ever be able to do ‘enough’ to subjectively correct the perceived inequity. Instead, beliefs associated with the need to repair can be identified and addressed. If the appraisals linked to their survival move from negative (e.g. ‘I survived in their place’) to more neutral (e.g. ‘it was just chance’), then the repair instinct should reduce.

Clients can be encouraged to engage in positive activities, rather than repair activities, which are linked to new beliefs about survival. For example, where a belief has been accessed such as ‘he would want me to be happy’, time can be taken in therapy to help clients to consider what is meaningful for them in their life and to devote time to it, as well as making plans for the future (similar to the ‘reclaiming your life’ intervention in cognitive therapy for PTSD; Ehlers & Clark, 2000). Here the emphasis is not on doing things because you ‘owe’ it to the deceased, nor about forgetting the trauma or those who died, but on finding a meaningful way to live your best life.

### 6 Processing intrusions

For individuals who are experiencing PTSD as well as survivor guilt, evidence-based treatments for PTSD should be delivered (trauma-focused CBT or EMDR are recommended in most guidelines e.g. NICE, 2018). In cognitive therapy, trauma memories are addressed through imaginal reliving or narrative writing to identify the worst moments in the trauma memory (‘hotspots’) and their idiosyncratic meaning to the individual, and then ‘updated’ by introducing new meanings (Ehlers & Clark, 2000). Where one of the emotions linked to a hotspot is survivor guilt, this approach would allow for the consideration of appraisals related to survival, and the integration of this information back into the trauma memory. For example, one of our clients survived the sinking of a migrant boat while crossing the Mediterranean sea, which killed many others, including his father. He experienced intrusive PTSD re-experiencing symptoms, including flashbacks and nightmares, to the event. Several hotspots in the trauma memory related to survivor guilt appraisals, so updating information was discussed and introduced to the memory (e.g. ‘we all knew we were taking a risk, and could drown; my father would be happy that I survived and made it to Europe’).

Others who experience survivor guilt and do not have PTSD may still experience intrusive imagery, for example memories of the deceased when they were alive, their funeral, or constructed images. Again, there may be important meanings related to these images. For example, one client had intrusive images of his deceased friend’s children crying at the funeral, which represented to him the belief that he should have died rather than his friend, as he was childless so would have been less missed. Some survivors report appraisals about the experience of having images (for example that they are going mad, are being haunted, or that the images mean the person is not forgotten), which can be addressed using guided discovery techniques to consider less threatening explanations. Imagery rescripting may be a useful technique to introduce new meanings into such memories. For example, a client whose father died in the early wave of the COVID-19 pandemic, when visitors were not allowed in hospitals, had a recurrent image of him in pain and alone, associated with the belief ‘he died alone and in agony, believing we didn’t care enough to be with him’. A new appraisal, developed through therapy that ‘he knew why we couldn’t be there, he knew that we loved him. He was with a nurse and was unconscious when he passed’ was represented in an imagery rescript where she imagined her father drifting painlessly away, comforted by a kind nurse, and then in heaven, happy and pain-free, watching over her with love and without blame.

## Limitations and areas for future research

The cognitive model and treatment recommendations outlined in this paper are yet to be empirically validated. They draw on existing theory and preliminary research into survivor guilt, as well as on established cognitive theory and therapies developed for similar problems. However, this is the first attempt to apply these ideas to the problem of survivor guilt, and both the model and treatment suggestions require evaluation.

Future research should focus on testing the various hypotheses generated by the model, including the risk factors, maintenance cycles and proposed development of the problem. Further work with a survivor population may also reveal other important mechanisms in understanding guilt. Research exploring cross-cultural variations and applicability of the model may be particularly important. The model outlined in this paper is viewed as a working model, not a final one. As well as stimulating debate and research in this neglected topic, our other goal is to develop an effective treatment approach to help individuals struggling with survivor guilt. This will require systematic testing and refining of the suggested treatment interventions described here, and others which may prove useful. Another area of further research would also concern the development of a scale to measure survivor guilt, which will prove necessary for evaluation of treatment attempts.

## Data Availability

Data availability is not applicable to this article as no new data were created or analysed in this study.

## Key learning aims

- To appreciate an overview of the current available literature on the phenomenology and prevalence of survivor guilt
- To understand a preliminary cognitive conceptualisation of survivor guilt
- To understand and be able to implement treatment recommendations for addressing survivor guilt

## Key practice points

- Survivor guilt is a common experience after traumatic events in which others have died and so requires assessment in survivors of traumas in which others died
- A cognitive conceptualisation of survivor guilt centres on an appraisal of unjust inequity, that the survivor has unfairly benefitted, and is maintained via rumination, secondary appraisals, intrusive imagery and thwarted attempts to repair
- Interventions to address the inequity appraisal are used to help the client access alternative explanations for their survival, and maintenance processes are also targeted
